# Both mass ratio effects and community diversity drive biomass production in a grassland experiment

**DOI:** 10.1038/s41598-018-37190-6

**Published:** 2019-02-12

**Authors:** Judit Sonkoly, András Kelemen, Orsolya Valkó, Balázs Deák, Réka Kiss, Katalin Tóth, Tamás Miglécz, Béla Tóthmérész, Péter Török

**Affiliations:** 1 MTA-DE Lendület Functional and Restoration Ecology Research Group, Egyetem tér 1, Debrecen, H-4032 Hungary; 2 University of Debrecen, Department of Ecology, Egyetem tér 1, Debrecen, H-4032 Hungary; 3 MTA-DE Biodiversity and Ecosystem Services Research Group, Egyetem tér 1, Debrecen, H-4032 Hungary; 4 MTA’s Premium Postdoctoral Research Programme, MTA TKI Nádor utca 7, Budapest, H-1051 Hungary

## Abstract

The maintenance of biodiversity is crucial for ecosystem processes such as plant biomass production, as higher species richness is associated with increased biomass production in plant communities. However, the effects of evenness and functional diversity on biomass production are understudied. We manipulated the composition of an experimental grassland by sowing various seed mixtures and examined the effects of diversity and evenness on biomass production after three years. We found that biomass production increased with greater species and functional richness but decreased with greater species and functional evenness. Standing biomass increased but species number and functional richness decreased with increasing proportion of perennial grasses. Our findings emphasise the importance of productive dominant species, as the proportion of perennial grasses had a positive effect on standing biomass, while species and functional evenness had a negative effect on it. Thus, our findings support the theory that, besides diversity, dominance effects and the so-called mass ratio hypothesis may also play a key role in explaining primary biomass production.

## Introduction

Habitat fragmentation, degradation, overexploitation and biological invasions are causing a global decline in biodiversity^1^. The maintenance of biodiversity is crucial, as it influences ecosystem functioning. For example, higher biodiversity in general is predicted to increase the biomass production and stability of ecosystems, and to decrease their invasibility^2^. Thus, understanding the consequences of the widespread loss of biodiversity has become one of the most important tasks of ecological research^3,4^. Energy enters into terrestrial ecosystems mostly by means of plant biomass production; thus, the impact of global biodiversity loss on plant production indirectly affects all ecosystem processes and associated services provided to humanity^5,6^.

The upper limits of primary production are clearly determined by the abiotic environment, but biotic characteristics such as biodiversity also shape biomass production^6,7^. One of the most debated questions in ecology is whether biomass production affects plant biodiversity or vice versa^8–10^. In initial observational studies of mature natural communities^11,12^ results usually showed a unimodal (hump-shaped) relationship between biomass production and diversity^9^. Namely, with increasing plant production diversity first increases, then after a certain point starts to decrease, as communities with high biomass are usually dominated by highly productive competitive species^13,14^. The unimodal relationship with plant productivity has also been demonstrated for strategy richness and for the variance of many traits^15^. However, the existence of a unimodal relationship between plant biomass production and diversity is debated^8,16^. More recent studies have presented primary production as the dependent rather than the independent variable^6,17^, usually by demonstrating that biomass production generally increases with plant diversity in newly established experimental communities^18,19^. One of the most important conclusions of these studies is that although high community biomass production tends to decrease biodiversity, the opposite, namely that decreasing diversity generally increases biomass production, cannot be inferred^6^. Later studies have linked these opposing viewpoints by emphasising that community biomass and diversity are both influenced by the resource supply rate of the habitat^10,20^.

Three main potential explanations exist for the positive relationship between biodiversity and biomass production observed in experimental studies. Firstly, the so-called sampling or selection effect suggests that a more diverse community has a higher probability of including highly productive species^21^. Secondly, complementarity effects occur when differences in species resource acquisition in space and time allow a more complete utilization of resources, resulting in the increased biomass production of more diverse communities^22^. Thirdly, facilitative effects of some co-occurring species can also positively influence biomass production by allowing the establishment and survival of other species^23–25^. Studies found sampling effects to be of greater importance than complementarity effects^26,27^; however, biodiversity effects cannot be explained solely by sampling effects, which occur far less generally than previously thought^28,29^. Sampling, complementarity and facilitative effects are mutually non-exclusive and the net effect of biodiversity on biomass production can be seen as the sum of these three effects^7^.

Linking diversity with ecosystem functions by niche complementarity is based on the distribution of species with different traits in the niche space, i.e. functional diversity^30,31^. There is growing awareness that besides species diversity functional diversity and composition (functional group and/or functional trait composition and diversity) are also important drivers of biomass production and may be more strongly linked to biomass production than species richness per se^32,33^. Considering functional diversity can contribute to our understanding of the consequences of biodiversity loss upon ecosystems^34^. There is considerable evidence for the importance of functional group richness and composition^18,35,36^, and also functional diversity and composition^37^. Further investigations of the interrelations of species and functional diversity, community structure and biomass production are necessary to advance understanding of biodiversity effects and the underlying processes^33^. As environmental and habitat conditions likely drive functional diversity, the effects of variation in functional diversity should be studied in a given vegetation type to provide meaningful comparisons.

Evenness measures how equally abundances – most frequently expressed as surface cover and/or biomass – are distributed among species and is inversely related to dominance. Effects of evenness on biomass production are far less studied than the effects of species and functional richness^38^. Some studies have demonstrated that evenness has a positive effect on biomass production^32,39,40^. Similarly, Nijs & Roy^41^ predicted that evenness should have a positive linear effect on biomass production. However, negative effects of evenness have also been demonstrated^42^, as a community may be most productive when it is dominated by a highly productive species^43^. Similarly, the mass ratio hypothesis^44^ proposes that the rate of an ecosystem function such as biomass production is primarily determined by the traits of the dominant species. Most biodiversity experiments used unnaturally high evenness levels^45,46^ (but see van Rooijen *et al*.^47^), further underlining the need to study the effect of evenness on biomass production in natural ecosystems^41,43^.

The results of previous biodiversity experiments were inconclusive about the effects of evenness and functional diversity on biomass production and further insights into the exact role of species diversity in determining community biomass production are also needed. Thus, we aimed to study the above-mentioned effects in experimental grasslands. To this end, we directed the development of grasslands established on former channels immediately after filling them up with soil by sowing seed mixtures with different grass/forb ratio. We hypothesised that (i) standing biomass is positively correlated with species and functional diversity. In line with the mass ratio hypothesis^44^, we expected that the abundance of the perennial grass functional group has a crucial role in determining both biomass production and diversity. Thus, we also hypothesised that (ii) the proportion of perennial grasses’ biomass is positively correlated to standing biomass, but negatively correlated to species diversity, evenness, and functional diversity.

## Methods

### Study area

The study site was in the Hortobágy National Park in East-Hungary, near the settlement Tiszafüred. The national park is characterised by open habitats, such as alkali grasslands, marshes and loess steppes^48^. The region has a moderately continental climate, with a mean annual precipitation of 550 mm and mean annual temperature of 9.5 °C, but with high variability between years^49^.

The drainage and watering channels established in the area in the 1950s and ‘60s altered the natural water regime of the region and lowered the groundwater table^50^. In response, several landscape-scale restoration projects were initiated aiming to eliminate these channels^48^. The experiment was located in the Villongó area (47°34′, 20°59′), within an approximately 100-ha area, which is characterised by Cynodonti-Poetum angustifoliae loess pastures^51,52^. Channels in the Villongó area were filled in October 2012, using the soil of the channel embankments, which were built from the excavated soil. After filling up the channels and levelling the surface, no other restoration measures were applied. The whole area was subsequently subject to low-intensity sheep grazing. Data on the composition of the surrounding vegetation is in Supplementary Dataset 1.

### Treatments and sampling

To generate an experimental gradient in diversity and evenness we used seed sowing treatments (Table 1). We used a randomized block design with three replicates, each of which contained five 5 m × 5 m plots along the channel. Instead of directly manipulating species numbers, the treatments were designed to manipulate the ratio of grasses and forbs (Table 1). We randomly assigned one of the five treatments to plots in each block. The sown species were selected from grassland-specialist species frequently occurring in the region of the study. We collected seeds in 2012 from natural populations of several locations in the study region and sowed them in late October 2012 (for sown species and seed amounts see Table 1). Seeds were sown on bare ground after filling up the channels with soil. No other pioneer vegetation was present on the site of the experimental plots. This way we directly manipulated initial community composition under uniform environmental conditions. After seed sowing, vegetation development was not manipulated, no weeding and no further treatment was applied, to let natural colonisation and assembly processes occur^53^.

**Table 1:** Thousand-seed mass (TSM) of the sown species and the total mass and number of seeds sown in each treatment.

		Treatments				
	TSM (g)	Forb	G5F25	G15F15	G25F5	Grass
Grass sowing (kg/ha)		—	5	15	25	30
Forb sowing (kg/ha)		30	25	15	5	—
Grass sowing (seed/m^2^)*		—	1500	4400	7300	8800
Forb sowing (seed/m^2^)*		5200	4300	2600	900	—
**Grass sowing**						
*Festuca rupicola*	0.342	—	1460	4382	7304	8764
**Forb sowing**						
*Achillea collina*	0.052	939	783	470	157	—
*Bunias orientalis*	33.444	10	8	5	2	—
*Carthamus lanatus*	15.141	5	4	2	1	—
*Centaurea jacea* ssp. *angustifolia*	1.055	8	7	4	1	—
*Centaurea scabiosa*	4.859	95	79	47	16	—
*Centaurea solstitialis*	1.162	68	57	34	11	—
*Filipendula vulgaris*	0.681	183	153	92	31	—
*Galium verum*	0.337	906	755	453	151	—
*Hypericum perforatum*	0.108	1432	1194	716	239	—
*Knautia arvensis*	3.137	18	15	9	3	—
*Lotus corniculatus*	1.091	33	27	16	5	—
*Lycopsis arvensis*	6.109	7	6	3	1	—
*Pimpinella saxifraga*	0.567	285	238	143	48	—
*Plantago media*	0.240	341	284	170	57	—
*Rapistrum perenne*	6.677	58	49	29	10	—
*Salvia nemorosa*	0.842	643	536	322	107	—
*Silene vulgaris*	0.703	132	110	66	22	—

Seed numbers were calculated using the sown mass and the thousand-seed mass of the species. *Rounded to the nearest hundred. Notations: ‘G’ and ‘F’ in treatment names stand for ‘Grass’ and ‘Forb’, respectively.

We focused on aboveground standing biomass as a proxy of plant biomass production, as there is a sigmoidal relationship between aboveground standing biomass and net primary productivity across a wide range of plants and habitats^54^. We collected biomass samples in late May 2015, near the peak of standing biomass in the region^13^. We collected total aboveground biomass from 12 quadrats (20 cm × 20 cm) from every 5 m × 5 m plot; thus, a total of 180 samples were collected (12 quadrats × 5 treatments × 3 experimental blocks). To avoid edge effects, biomass samples were collected from the inner 4 m × 4 m area of each plot. We then dried samples at 65 °C (24 h) and sorted them to vascular plant species and litter. We measured the dry mass of the sorted species and litter with an accuracy of 0.01 g. Nomenclature follows Király^55^.

### Statistical analyses

As we did not remove the spontaneously colonising species and the experimental communities contained a considerable amount of non-sown species, we calculated all measures (both standing biomass and all diversity measures) considering both the sown and the spontaneously colonising species together. We used species richness and species evenness as species diversity measures, calculated for each plot considering both the sown and the spontaneously colonising species. As suggested by Mouchet *et al*.^56^, we calculated three functional diversity indices, functional richness (FRic), functional evenness (FEve) and functional divergence (FDiv), as these constitute a relevant combination that considers different facets of functional diversity and were built to be complementary^57^. We calculated these indices using LHS traits (leaf-height-seed traits, i.e. SLA, canopy height and seed mass, as proposed by Westoby^58^), which capture basic processes of plant functioning^26^. Trait data for the studied species are available in Supplementary Dataset 2. For the calculation of FRic, FEve and FDiv indices we used the FD library^59^ in R statistical environment^60^. We obtained canopy height and seed mass data from sources containing regionally measured data^55,61,62^, and SLA data from the LEDA Traitbase^63^. The proportion of perennial grasses’ biomass (PG%) was calculated as the proportion of perennial grasses’ biomass to the total biomass in each plot. Species number, species evenness and functional diversity indices (FRic, FEve and FDiv) were calculated for the biomass sample size (0.04 m^2^).

We used linear mixed models (LMMs) to test the effect of species number, species evenness and functional diversity (FRic, FEve, FDiv) on the standing biomass of plots, with experimental block included as a random factor. We also used LMMs to test the effect of PG% on the species number, species evenness and functional diversity (FRic, FEve, FDiv) of plots. LMMs were performed using the ‘lmer’ function in the package ‘lme4’^64^ in R. In order to achieve normally distributed residual errors some of the variables (standing biomass and FRic) were log transformed, and species number was standardized using the ‘scale’ function in R. Measures for the different facets of biodiversity were either weakly correlated to each other or not correlated, the strongest correlation was observed between species richness and FRic (r = 0.705), which is on the threshold of considering two variables non-collinear^65^ (Table S1 in Supplementary Table). Marginal and conditional R^2^ values were obtained using the function ‘r.squaredGLMM’ provided in the package ‘MuMIn’^66^. Marginal R^2^ is the proportion of variance explained by the fixed effects, while conditional R^2^ is the proportion explained by the full model, including both fixed and random effects^67^.

As the visual inspection of species evenness plotted against PG% clearly indicated a unimodal relationship, we fitted both a linear and a quadratic model to the data and compared them using the Akaike Information Criterion (AIC)^68,69^, where smaller AIC values indicate better models. We considered a difference in AIC values higher than 2 (ΔAIC > 2) to indicate moderate support for a difference between the models, while ΔAIC > 10 to indicate strong support for a difference^69^. All statistical analyses were carried out in R statistical environment^60^.

## Results

We found a total of 83 species in the plots, of which 68 species were non-sown. The mean number of species per 5 m × 5 m plot was 34.1 (min = 24, max = 52), of which a mean of 6.5 species were sown (min = 2, max = 13), and a mean of 27.5 species were non-sown (min = 20, max = 40). Thus, the composition and diversity of the plots after 3 years were considerably different from those of the sown mixtures. *Festuca rupicola* was the most abundant in the majority of plots where it was sown, but other perennial grasses (*Agropyron repens*, *Agrostis stolonifera*, *Alopecurus pratensis*, *Cynodon dactylon*, *F. pseudovina*, *Koeleria cristata*, *Poa angustifolia*, *P. bulbosa* and *P. pratensis*) colonised the plots spontaneously. Where no grass was sown, mostly the short-lived grass *Bromus mollis* or the perennial grass *Poa angustifolia* was the most abundant. In some plots sown dicot species (*Achillea collina*, *Hypericum perforatum*, *Plantago media* or *Salvia nemorosa*) were the most abundant. Species’ biomass data of all the plots are in Supplementary Dataset 3.

**Figure 1:**
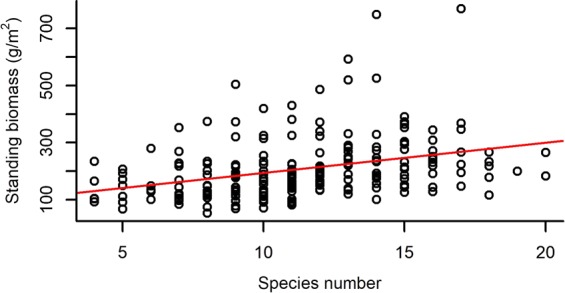
The relationship of species number and standing biomass (for the results of LMMs see Table 2). Marginal R^2^ = 0.179 (the proportion of variance explained by the fixed effects).

**Figure 2:**
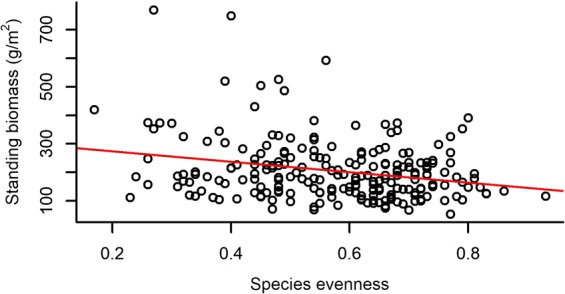
The relationship of species evenness and standing biomass (for the results of LMMs see Table 2). Marginal R^2^ = 0.066 (the proportion of variance explained by the fixed effects).

**Figure 3:**
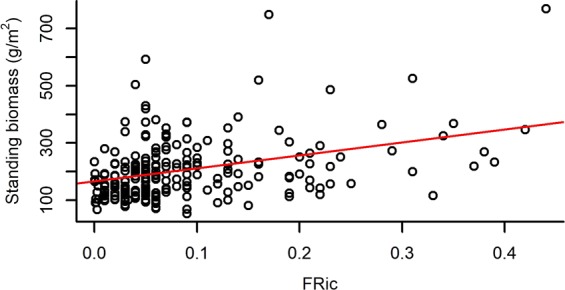
The relationship of functional richness (FRic) and standing biomass (for the results of LMMs see Table 2). Marginal R^2^ = 0.148 (the proportion of variance explained by the fixed effects).

**Figure 4:**
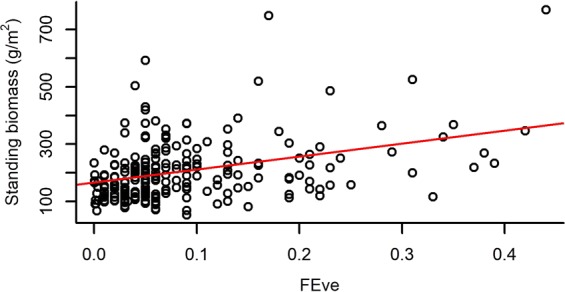
The relationship of functional evenness (FEve) and standing biomass (for the results of LMMs see Table 2). Marginal R^2^ = 0.053 (the proportion of variance explained by the fixed effects).

**Figure 5:**
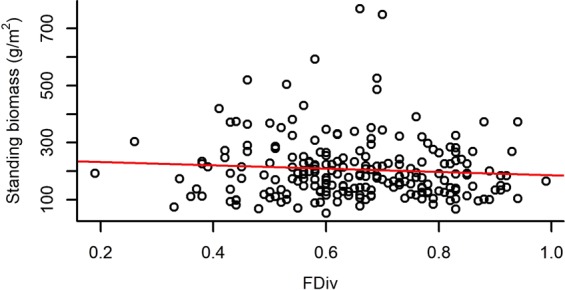
The relationship of functional divergence (FDiv) and standing biomass (for the results of LMMs see Table 2). Marginal R^2^ = 0.035 (the proportion of variance explained by the fixed effects).

Both species richness and evenness, and functional diversity (FRic – Functional richness; FEve – Functional evenness; FDiv – Functional divergence) had a significant effect on the standing biomass of plots (Table 2). Species richness and FRic had a relatively strong positive effect on standing biomass, while species evenness, FEve and FDiv had weak negative effects on it (Figs 1–5).

**Table 2:** The effect of different community characteristics on standing biomass.

	*χ* ^*2*^	df	*P*	marginal R^2^	conditional R^2^
Species number	37.318	1	<0.0001	0.179	0.197
Species evenness	12.582	1	<0.0001	0.066	0.083
FRic	31.480	1	<0.0001	0.148	0.169
FEve	10.484	1	0.001	0.053	0.102
FDiv	6.888	1	0.009	0.035	0.065

The effect of every variable was tested with linear mixed models. Marginal R^2^ is the proportion of variance explained by the fixed effects, while conditional R^2^ is the proportion explained by the full model, including both fixed and random effects. Notations: FRic – Functional richness, FEve – Functional evenness, FDiv – Functional divergence.

As the visual inspection of species evenness plotted against the proportion of the perennial grasses’ biomass (hereafter PG%) indicated a unimodal relationship, we fitted both a linear (*χ*^*2*^ = 17.099, Df = 1, *P* < 0.0001) and a quadratic model (*χ*^*2*^ = 126.14, Df = 2, *P* < 0.0001) to the data. The high difference in the AIC values of the two models (ΔAIC = 78.57; AIC = −175.92 and AIC = −254.49, respectively) strongly supported that the relationship of species evenness and PG% is unimodal.

**Figure 6:**
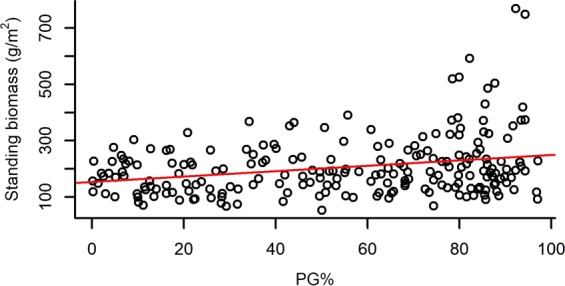
The relationship of the proportion of perennial grasses’ biomass to total biomass (PG%) and standing biomass. (For the results of LMMs see Table 3). Marginal R^2^ = 0.034 (the proportion of variance explained by the fixed effects).

**Figure 7:**
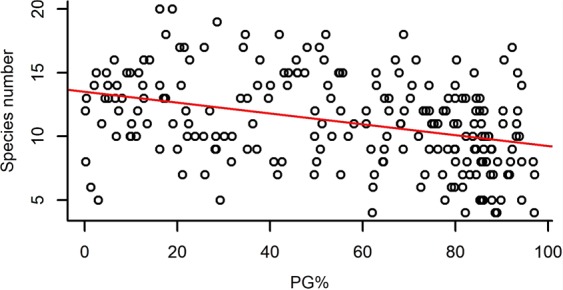
The relationship of the proportion of perennial grasses’ biomass to total biomass (PG%) and species number. (For the results of GLMMs see Table 3). Marginal R^2^ = 0.130 (the proportion of variance explained by the fixed effects).

**Figure 8:**
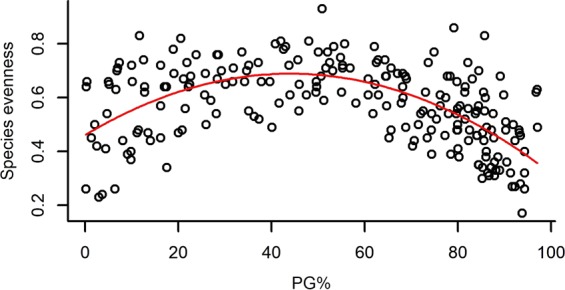
The relationship of the proportion of perennial grasses’ biomass to total biomass (PG%) and species evenness with the fitted second order polynomial curve. (For the results of LMMs see Table 3). Marginal R^2^ = 0.392 (the proportion of variance explained by the fixed effects).

**Figure 9:**
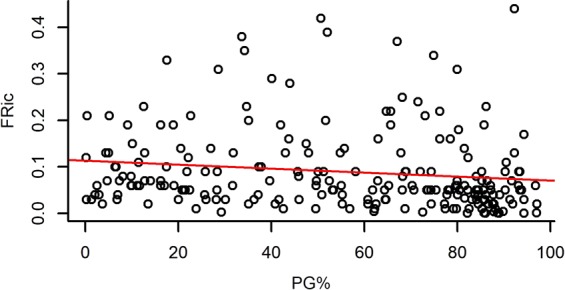
The relationship of the proportion of perennial grasses’ biomass to total biomass (PG%) and functional richness (FRic) (For the results of GLMMs see Table 3). Marginal R^2^ = 0.061 (the proportion of variance explained by the fixed effects).

**Figure 10:**
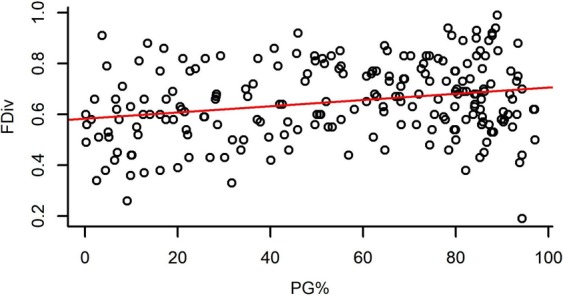
The relationship of the proportion of perennial grasses’ biomass to total biomass (PG%) and functional divergence (FDiv) (For the results of LMMs see Table 3). Marginal R^2^ = 0.027 (the proportion of variance explained by the fixed effects).

PG% ranged from 0.16% to 97.05% with a mean of 55.12%, and it had a significant effect on every studied variable except for FEve (Table 3). Higher PG% had a slight positive effect on standing biomass and FDiv, whereas it had a relatively strong negative effect on species number and FRic and showed a notably strong unimodal relationship with species evenness (Figs 6–10).

**Table 3:** The effect of the proportion of perennial grasses’ biomass (PG%) on community characteristics.

	*χ* ^*2*^	df	*P*	marginal R^2^	conditional R^2^
Standing biomass	6.4004	1	0.011	0.034	0.076
Species number	29.728	1	<0.0001	0.130	0.235
Species evenness^2^	126.14	2	<0.0001	0.392	0.443
FRic	12.301	1	0.0005	0.061	0.132
FEve	0.323	1	0.570	—	—
FDiv	5.004	1	0.025	0.027	0.027

Notations: FRic – Functional richness, FEve – Functional evenness, FDiv – Functional divergence.

The effect of PG% on every variable was tested with linear mixed models. Marginal R^2^ is the proportion of variance explained by the fixed effects, while conditional R^2^ is the proportion explained by the full model, including both fixed and random effects.

As PG% showed a unimodal relationship with species evenness, we tested whether this was due to negative correlation between the abundance of annual grass species and the abundance of perennial grasses; i.e. that with low abundance of perennial grasses, the high abundance of annual grasses causes low species evenness. We found that the proportion of annual grasses’ biomass to total biomass (AG%) is indeed strongly negatively correlated to PG% (Spearman rank correlation, rho = −0.761, *P* < 0.0001, see Fig. S1 in Supplementary Figures), and that AG% also shows a humped-back relationship with species evenness (second order polynomial model, R^2^ = 0.337, *P* < 0.0001, see Fig. S2 in Supplementary Figures).

## Discussion

The net effect of biodiversity on biomass production can be seen as the sum of sampling, complementarity and facilitative effects^7^, usually indicating a positive relationship between diversity and biomass production^27,36,70^, especially at a constant resource level^71^. Thus, our finding that species number was significantly positively correlated with standing biomass is in line with our first hypothesis and most prior studies. Roscher *et al*.^72^ found a significant relationship between total biomass production and total species richness (i.e. sown and spontaneously colonising species) both in weeded and non-weeded plots, but the relationship disappeared in the non-weeded plots when only the sown species were taken into account. This result is in accordance with our finding that total aboveground biomass is significantly affected by total species richness (considering both sown and colonising species) without weeding the plots. Conversely, no or weak effect of species richness on biomass production has also been reported^37,73^. Others have stated that although mixtures produce more biomass than monocultures, this effect reaches a limit at very low species richness. For example, Roscher *et al*.^74^ found that the positive effect of species richness was limited at 2 or 3 species (compared to monocultures). In contrast, in our case the positive effect of species richness on biomass production could be detected even at much higher species numbers (see Fig. 1).

Among our most interesting findings was the negative relationship between standing biomass and both species and functional evenness (FEve). Both niche complementarity and facilitative effects can be the strongest at high evenness levels^75,76^, as evenness determines the relative importance of inter- vs. intraspecific interactions^45^. Intraspecific competition is expected to be more intense than interspecific competition and the increased level of species evenness (i.e. lower variance in the abundances of different species) is usually accompanied by the decreased level of intraspecific competition^77^. Moreover, high functional evenness indicates effective resource utilization, which supports the development of productive communities^57,78^. Consequently, evenness is generally expected and reported to be positively correlated to biomass production in plant comminites^39,41,79^. On the contrary, the mass ratio hypothesis predicts that the rate of an ecosystem process (such as biomass production) is mostly determined by the identity and traits of dominant species^44^. Namely, considering uniform environmental conditions, a community can have the highest biomass production if it is strongly dominated by a highly productive species, from which it can be inferred that evenness is negatively correlated with biomass production^43^. Accordingly, some studies have found negative^42^ or no relationship^75^ between species evenness and biomass production. Vile *et al*.^80^ found that the biomass production of a community can be predicted from the potential relative growth rate of species weighted by their abundances, which is in line with the mass ratio hypothesis. Thus, our result that species and functional evenness both had a negative effect on standing biomass is also in line with the mass ratio hypothesis. The fact that the proportion of perennial grasses’ biomass (PG%) had a positive, although weak effect on total standing biomass, meaning that plots with a high abundance of perennial grass species were the most productive, also corroborates the mass ratio hypothesis. The negative effect of functional divergence (FDiv) on standing biomass may further strengthen this relationship, possibly indicating that the trait values of perennial grasses are close to the centre of the community trait space; thus, high abundance of these species causes low FDiv.

The perennial grass functional group played a key role in determining not only biomass production, but also species diversity, species evenness and functional richness (FRic) and functional divergence (FDiv). In line with our second hypothesis, species number and functional richness decreased with the increasing abundance of perennial grasses, but species evenness and PG% showed a humped-back relationship. As we demonstrated, this was caused by the abundance of perennial grasses (mostly *Festuca rupicola*, *F. pseudovina* and *Poa angustifolia*) being strongly negatively correlated to the abundance of annual grasses (mostly *Bromus mollis*). Thus, the high abundance of annual grasses resulted in low evenness in plots with a low abundance of perennial grasses, while evenness reached the highest values in plots where neither perennial nor annual grasses had high abundance. In this case annual grasses such as *Bromus mollis* could take over the role of perennial grasses in the community regarding biomass production.

The fact that species and functional richness (FRic) were positively correlated to standing biomass emphasises the importance of biodiversity in the maintenance of primary production and demonstrates that complementary effects probably influenced biomass production. Although complementarity effects are usually considered to be of more importance than sampling effects^29,81^, some studies found biodiversity effects to be more strongly associated with sampling or dominance effects than with complementarity^48,82^. In our experiment, sampling effects may have been more important in determining biomass production than complementarity effects, because species and functional evenness were negatively correlated with community biomass and plots with a high abundance of productive perennial grasses generally had more biomass.

In field studies it is rather difficult to find a wide range of diversity under homogeneous environmental conditions. Thus, field studies without experimental manipulation of diversity are usually confounded by differing environmental conditions^5,35^. On the contrary, greenhouse and mesocosm experiments have the advantage of uniform conditions, but they usually use species numbers that are considerably lower than in a natural community^39,81,83^, and because of the small scale of the experiments the validity of the results to natural ecosystems is limited. We have overcome these obstacles by experimentally manipulating diversity and evenness under homogeneous field conditions. This way, the range of diversity was broad enough with uniform environmental conditions. Thus, the scale of ecological processes was also more meaningful for natural ecosystems^35,84^.

Biodiversity is likely to determine ecosystem functions more strongly when positive interactions among species (facilitation or complementary resource use) drive the function in question, such as biomass production. In such cases decreased dominance and increased evenness can result in the increase of this function^43^. On the contrary, if the identity and traits of the dominant species are more important for an ecosystem process such as biomass production, strong dominance accompanied by low evenness can result in higher biomass production^85,86^. In this case it seems that both diversity and dominance effects are important determinants of biomass production. Our results emphasise that although facilitative and complementarity effects can play a key role in determining ecosystem functions, thus conserving biodiversity is indispensable to maintain them, we should not underestimate the importance of dominance effects.

## Supplementary information

Supplementary Figures

Supplementary Table

Supplementary Dataset 1

Supplementary Dataset 2

Supplementary Dataset 3

## Data Availability

All data are available in the Supplementary Datasets.
