# Supplementary material for: Both mass ratio effects and community diversity drive biomass production in a grassland experiment
Source: Sci Rep. 2019 Feb 12;9:1848. doi: 10.1038/s41598-018-37190-6 (PMC6372655; doi:10.1038/s41598-018-37190-6)
Supplement: Supplementary file 1 — Supplementary Figures [file 41598_2018_37190_MOESM1_ESM.pdf]

Both mass ratio effects and community diversity drive biomass production in a grassland experiment

Judit Sonkoly, András Kelemen, Orsolya Valkó, Balázs Deák, Réka Kiss, Katalin Tóth, Tamás Miglécz, Béla Tóthmérész & Péter Török

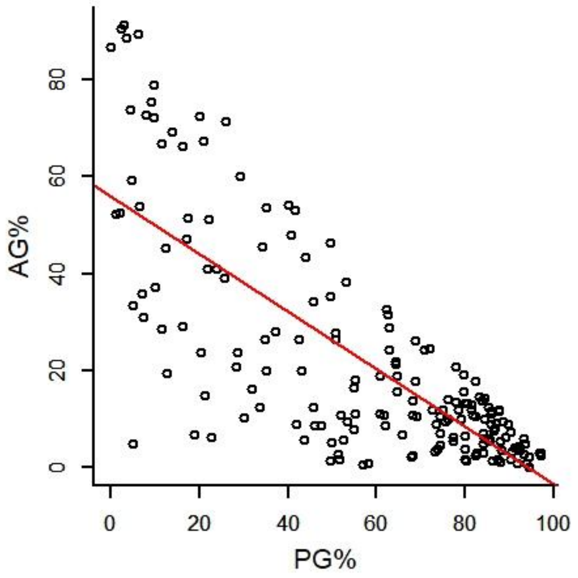

Figure S1. The relationship of the proportion of annual grasses' biomass (AG%) and perennial grasses' biomass (PG%) (Spearman rank correlation, rho=-0.761).

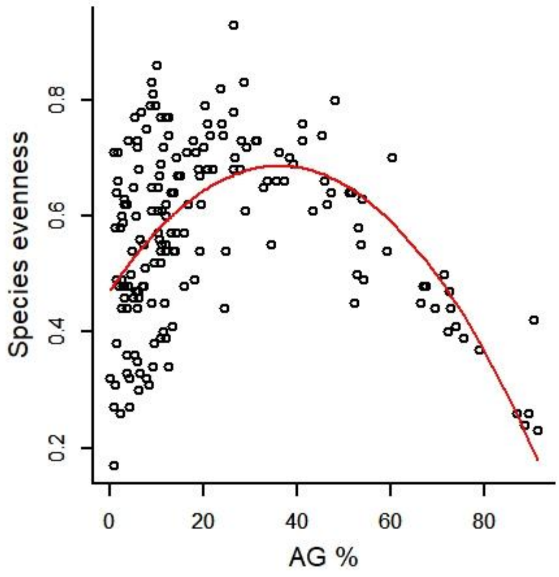

Figure S2. The effect of the proportion of annual grasses' biomass (AG%) on species evenness (second order polynomial model, R^2=0.337).
