# Supplementary material for: Both mass ratio effects and community diversity drive biomass production in a grassland experiment
Source: Sci Rep. 2019 Feb 12;9:1848. doi: 10.1038/s41598-018-37190-6 (PMC6372655; doi:10.1038/s41598-018-37190-6)
Supplement: Supplementary file 2 — Supplementary Table [file 41598_2018_37190_MOESM2_ESM.pdf]

Both mass ratio effects and community diversity drive biomass production in a grassland experiment

Judit Sonkoly, András Kelemen, Orsolya Valkó, Balázs Deák, Réka Kiss, Katalin Tóth, Tamás Miglécz, Béla Tóthmérész & Péter Török

Table S1. Correlations between the different facets of biodiversity (Pearson correlation, significant results are marked with bold).

	Species richness	Evenness	FRic	FEve
**Evenness**	p=0.085			
**FRic**	**p<2.2e-16 r=0.705**	p=0.083		
**FEve**	p=0.399	p=0.226	**p=0.011 r=-0.173**	
**FDiv**	p=0.253	p=0.374	**p=0.039 r=-0.141**	p=0.143
